# Effect of Rehabilitation with a New Ocular Prosthesis on Electromyography of the Occipitofrontalis, Temporal, Masseter, and Sternocleidomastoid

**DOI:** 10.1055/s-0041-1735795

**Published:** 2021-11-22

**Authors:** Fernanda Pereira de Caxias, Karen Letícia Sa Teles, Daniela Micheline dos Santos, Clóvis Lamartine de Moraes Melo Neto, Emily Vivianne Freitas da Silva, Marcelo Coelho Goiato, André Pinheiro de Magalhães Bertoz, Marcella Santos Januzzi, Karina Helga Turcio

**Affiliations:** 1 Department of Dental Materials and Prosthodontics, School of Dentistry, São Paulo State University, Araçatuba, São Paulo, Brazil; 2 Oral Oncology Center, School of Dentistry, São Paulo State University, Araçatuba, São Paulo, Brazil; 3 Department of Pediatric and Social Dentistry, School of Dentistry, São Paulo State University, Araçatuba, São Paulo, Brazil

**Keywords:** ocular prosthesis, facial muscles, electromyography, eye, temporal muscle, masseter muscle

## Abstract

**Objectives** The aim of this study was to verify during facial expressions (“happy,” “sad,” “fearful,” “angry,” “surprised,” and “disgusted”) if: (1) there would be difference in the electromyography (EMG) of the occipitofrontalis, temporal, masseter, and sternocleidomastoid muscles on the normal side (NS) compared with the affected side (AS) (without the use of an ocular prosthesis) in individuals with unilateral absence of the eyeball, and (2) the rehabilitation with a new ocular prosthesis would affect the EMG of the muscles studied on the AS in these individuals.

**Materials and Methods** Thirteen individuals, without temporomandibular disorder, with good health, with unilateral absence of the eyeball (the eye must have been removed by evisceration or enucleation), and users or nonusers of an ocular prosthesis were included. EMG of the occipitofrontalis, temporal, masseter, and sternocleidomastoid muscles was performed during rest and facial expressions (“happy,” “sad,” “fearful,” “angry,” “surprised,” and “disgusted”) before (T0) and 90 days after (T1) rehabilitation with a new ocular prosthesis. The analyses were performed in T0 on NS and AS (without the use of an ocular prosthesis), and in T1 on AS with the new ocular prosthesis.

**Statistical Analysis** All data were submitted to the Student's *t* -test with *p* < 0.05.

**Results** There was no statistically significant difference comparing the AS with the NS in T0 for all muscles studied, during all facial expressions evaluated ( *p* > 0.05). There was no statistically significant difference comparing the AS in T0 with itself in T1 for all muscles studied, during all facial expressions evaluated ( *p* > 0.05).

**Conclusion** Eye loss did not affect the EMG of studied muscles when comparing NS with AS (without the use of an ocular prosthesis). The rehabilitation with ocular prosthesis was not capable of changing the EMG on AS.

## Introduction

Movements and contractions of the orofacial muscles are managed by a complex cortical control that is subject to reflexive reactions, besides the emotional and volitional controls. 1 These controls depend on proprioception, which is a complex sensation that involves central and peripheral processes of information. These processes depend on rest and the movement which comes from the peripheral mechanoreceptors, which are centrally processed according to the corporal map. 2 3 Despite this motor complexity, the facial muscles contribute significantly to human behavior in many activities such as alimentation, production of speech, and visual communication for emotional states. 1

The mimic musculature is capable of performing more than 20 facial expressions, 4 including the basic expressions of happiness, fear, disgust, anger, surprise, and sadness. 4 These muscles have the main function of generating important facial expressions for nonverbal communication, 5 and consequently, for interpersonal relationships. The complexity of the trigeminal–cervical system has been related to pain. 6 However, in terms of motor control, a peculiarity of the facial musculature innervation is the presence of polyneural innervation, such as the presence of motor plates deriving from different motoneurons in different mimic muscles. 7 In addition, the polyneural innervation of facial muscles in adult humans also occurs in other muscles of the cranium, such as the larynx 8 and extraocular, 9 though the clinical significance is not known.

Based on the neurological and motor connection between different facial muscles and between facial and cervical muscles, it is important to verify the effect of an eye prosthesis on these muscles. Only two studies exist in the literature that electromyographically evaluate individuals that used an ocular prosthesis, 10 11 and none of these studies evaluated the occipitofrontalis, temporal, masseter, and sternocleidomastoid muscles. Therefore, the aim of this study was to verify during facial expressions (“happy,” “sad,” “fearful,” “angry,” “surprised,” and “disgusted”) if:

- There would be a difference between the electromyography (EMG) of occipitofrontalis, temporal, masseter, and sternocleidomastoid muscles on the normal side (NS) compared with the affected side (AS) (without the use of the ocular prosthesis) in individuals with unilateral absence of the eyeball by evisceration or enucleation. 12
- The rehabilitation with a new ocular prosthesis would affect the EMG of the muscles studied on the AS in these individuals.

## Materials and Methods

This study was approved by the Ethics Committee in Human Research of the São Paulo State University (UNESP), School of Dentistry (Araçatuba)—71062317.8.0000.5420. All procedures were carried out in accordance with the Declaration of Helsinki. 13

Patients with loss of one eye from the Oral Oncology Center (UNESP) were invited to participate in this study. After applying the inclusion and exclusion criteria, 21 patients were selected in this study, but only 13 of them participated. Then, the 13 participants signed an informed consent form.

### Inclusion Criteria

- Individuals included in this study were those with all of their teeth or close to complete dentition.
- Individuals who were submitted to evisceration or enucleation of only one eye, 12 with a normal contralateral eye.
- Users or nonusers of an ocular prosthesis and eligible for prosthetic rehabilitation.
- For those who were already using an ocular prosthesis—length of use of the old prosthesis must be at least 3 years.
- American Society of Anesthesiologists (ASA) 1 and ASA 2 (with controlled systemic diseases) individuals, according to the ASA classification. 14
- Individuals with cognitive ability to answer questions and follow instructions during examinations.

### Exclusion Criteria

- Individuals who were submitted to orbital exenteration. 15
- Individuals who were carriers of a serious illness such as trigeminal neuralgia, tumors, neurological illnesses, psychiatric problems, and narcolepsy.
- Individuals who used medication that could interfere with muscular activity (e.g., benzodiazepines). 16
- Abuse of alcohol and/or drug consumption.
- Individuals who presented a previous history of temporomandibular articulation surgery, degenerative illnesses, or neuropathic pain.
- Pregnancy.
- Individuals with facial paralysis.
- Individuals undergoing radiotherapy or chemotherapy treatment.
- Individuals with temporomandibular disorders confirmed through the Research Diagnostic Criteria for Temporomandibular Disorders questionnaire. 17
- Individuals with allergy to polymethylmethacrylate.

### Assessment Time Points

Two evaluations were performed, the first before rehabilitation with a new ocular prosthesis (T0) and the second, 90 days after rehabilitation (T1). EMG tests were performed on the occipitofrontalis, temporal, masseter, and sternocleidomastoid muscles.

- T0—Electromyographic examinations were performed on the NS and the AS (without the use of an ocular prosthesis) of the individuals.
- T1—A new EMG on the AS was performed with the individual using their new ocular prosthesis.

### Electromyographic Examinations

All individuals were instructed to wash the regions that would receive the electrodes with water and astringent soap. 18 Next, a soft rubbing was performed with gauze soaked in 70% alcohol, for the removal of oiliness from the skin, impedance reduction, and better signal conductivity. 11 18 The surface electromyographic signals were processed and visualized using the MyosystemBr1_P84 electromyograph and MyosystemBr1 3.5 software (DataHominis Tecnologia Ltda., Brazil). The configurations of the electromyograph connector were constant current tension output of ± 12 V at ± 100 mA and common-mode rejection ratio of 112 dB at 60 dB. It had protection against overloads and a low pass filter for elimination of noise from 5 Hz to 5 kHz.

For the occipitofrontal muscle, two electrodes (Meditrace 100, Covidien Ilc, United States) were placed on this muscle—the first electrode was placed just above the eyebrow, and from its center to the midline, there was a distance of 1.5 to 2 cm; in addition, an imaginary sagittal line connected the center of this electrode with the corner of the eye; the second electrode was placed just above the first, according to the orientation of the muscle fibers. For the masseter and temporal (anterior part of this muscle) muscles, the electrodes (Meditrace 100, Covidien Ilc) were positioned according to Goiato et al. 19 For the sternocleidomastoid muscle, two electrodes (Meditrace 100, Covidien Ilc) were placed on this muscle at a distance of 5 cm from the mastoid process, similar to the study by Guedes et al. 20 All electrodes were positioned bilaterally for each muscle studied.

The electrodes were made of polyethylene foam, Ag/AgCl double contact, and adherent hydrogel with low impedance. 18 After placing the electrodes, the electromyographic signals were tested and the gain was adjusted. The frequency of acquisition was 2,400 Hz, the filter was 1,000 Hz, and the electrode's gain was ×20.

During the entire electromyographic evaluation:

- The individuals were evaluated in a calm and silent environment. 11
- The individuals were seated comfortably in a chair, in an upright position, with their feet supported on the floor and their hands on their legs.
- All individuals remained with their heads in the same position, that is, the Frankfurt plane was parallel to the ground. 11
- The temperature of the enclosure was 23°C, so that the individuals did not perspire, avoiding the dislocation of the electrodes.
- The notebook and the electromyograph were not connected to an outlet, to avoid any type of influence on the collected data.

The recordings were done during rest and facial expressions (“happy,” “sad,” “fearful,” “angry,” “surprised,” and “disgusted”), 4 and each recording was made for 10 seconds. With the intention of standardizing the facial expressions which the individuals had to reproduce in each examination, photographs of the expressions were taken from the set of facial expressions developed by Du et al. 4 These photographs were printed in color using the normal quality setting on white paper, and a size of 12.5 cm × 9 cm. Therefore, the individuals were asked to perform the same expressions as in the photographs. 4

The electromyographic data were normalized 21 with the rest record of each muscle.

### Prostheses Fabrication

All the new ocular prostheses were fabricated according to the technique described by Goiato et al. 22 23 The acrylic resin in all cases was thermopolymerizable (Clássico, Brazil) using the conventional method (cycles of hot water).

Each patient was instructed on how to clean and disinfect their eye prosthesis. 24

## Statistical Analysis

The GPower 3.1 software (Heinrich-Heine-Universität Düsseldorf, Germany) was used for sample size estimation, which indicated that for the rest analysis, the N (number of participants) necessary would be eight participants (β = 0.2% and α = 0.05%).

MyosystemBr1 3.5 (DataHominis Tecnologia Ltda.) software was used to determine the root mean square value of the electrical signal (µV) obtained in the EMG tests. 18 The statistical analysis was performed by using the SPSS version 21.0 program (Statistical Package for the Social Sciences, IBM Corp, United States). The data of EMG were compared between the NS and the AS in T0, and for AF between T0 and T1. All data were submitted to the Student's *t* -test with a significance of 5%.

## Results

Twenty-one individuals were selected to participate in the study, but eight were excluded before conclusion (four did not return to receive their prostheses; one passed away; one needed reconstructive surgery; one did not use the prosthesis; and one did not want to keep participating in the study). Thus, 13 patients completed the two sessions with no dropouts or missing data. These patients were between 21 and 76 years old (average of 55 years), being seven men and six women. Regarding the history of eye surgery, five patients had undergone eviscerations and eight enucleations. Based on the patients included in this study, the time of eye loss ranged from 3 to 42 years (mean 15 years).

The Student's *t* -test demonstrated that there was no statistically significant difference comparing the AS with the NS in T0 ( Table 1 ) for all muscles studied, during all facial expressions evaluated ( *p* > 0.05). As well, Student's *t* -test demonstrated that there was no statistically significant difference comparing the AS in T0 with itself in T1 ( Table 2 ) for all muscles studied, during all facial expressions evaluated ( *p* > 0.05).

**Table 1:** Mean values (SD) of muscular electrical activity (µV) of occipitofrontalis, temporal, masseter, and sternocleidomastoid muscles of NS and AS during facial expressions before treatment (T0)

Muscle	Time points	Facial expressions					
		Happy	Fearful	Disgusted	Angry	Surprised	Sad
		Mean (SD)	Mean (SD)	Mean (SD)	Mean (SD)	Mean (SD)	Mean (SD)
Occipitofrontalis	NS	1.03 (0.29)	3.93 (4.02)	3.04 (1.99)	2.92 (1.56)	3.00 (2.19)	3.98 (7.27)
	AS	1.07 (0.35)	4.35 (3.56)	4.41 (3.93)	4.21 (3.46)	3.42 (2.61)	4.72 (6.96)
	*p* -Value	0.721	0.780	0.277	0.238	0.665	0.794
Temporal	NS	2.76 (1.57)	3.44 (2.18)	2.65 (1.42)	2.81 (2.63)	2.39 (1.37)	2.70 (2.14)
	AS	2.60 (1.54)	2.90 (1.76)	2.29 (0.93)	2.52 (1.75)	1.94 (0.72)	1.97 (1.22)
	*p* -Value	0.804	0.494	0.454	0.746	0.309	0.300
Masseter	NS	5.01 (3.87)	3.81 (3.22)	2.41 (1.52)	3.15 (2.79)	1.56 (0.97)	1.98 (1.12)
	AS	5.36 (4.70)	4.68 (4.60)	2.04 (0.91)	4.14 (4.26)	1.50 (1.06)	2.40 (2.38)
	*p* -Value	0.838	0.585	0.457	0.489	0.879	0.563
Sternocleidomastoid	NS	1.41 (1.00)	5.19 (6.03)	1.48 (1.27)	2.19 (2.71)	2.11 (2.34)	1.89 (1.84)
	AS	1.66 (1.33)	6.87 (9.50)	2.08 (2.20)	3.78 (7.72)	2.80 (2.89)	3.56 (5.80)
	*p* -Value	0.603	0.595	0.406	0.489	0.508	0.329

Abbreviations: AS, affected side; NS, normal side; SD, standard deviation.

Note: Student's *t* -test with 5% significance.

**Table 2:** Mean values (SD) of muscular electrical activity (µV) of occipitofrontalis, temporal, masseter, and sternocleidomastoid muscles of affected side during facial expressions before treatment (T0) and after 90 days of use of new prosthesis (T1)

Muscle	Time points	Facial expressions					
		Happy	Fearful	Disgusted	Angry	Surprised	Sad
		Mean (SD)	Mean (SD)	Mean (SD)	Mean (SD)	Mean (SD)	Mean (SD)
Occipitofrontalis	T0	1.07 (0.35)	4.35 (3.56)	4.41 (3.93)	4.21 (3.46)	3.42 (2.61)	4.72 (6.96)
	T1	1.17 (0.55)	5.39 (6.65)	5.26 (4.66)	7.20 (5.93)	3.78 (3.86)	5.46 (6.75)
	*p* -Value	0.524	0.479	0.556	0.115	0.778	0.757
Temporal	T0	2.60 (1.54)	2.90 (1.76)	2.29 (0.93)	2.52 (1.75)	1.94 (0.72)	1.97 (1.22)
	T1	2.88 (2.46)	4.69 (4.95)	3.84 (5.59)	2.63 (1.41)	2.07 (1.58)	2.71 (3.13)
	*p* -Value	0.718	0.201	0.332	0.828	0.783	0.365
Masseter	T0	5.36 (4.70)	4.68 (4.60)	2.04 (0.91)	4.14 (4.26)	1.50 (1.06)	2.40 (2.38)
	T1	6.63 (5.62)	5.73 (5.25)	3.16 (2.39)	6.14 (8.69)	1.96 (1.36)	3.33 (4.27)
	*p* -Value	0.509	0.351	0.084	0.360	0.204	0.249
Sternocleidomastoid	T0	1.66 (1.33)	6.87 (9.50)	2.08 (2.20)	3.78 (7.72)	2.80 (2.89)	3.56 (5.80)
	T1	2.93 (4.77)	6.77 (9.01)	3.00 (2.19)	6.03 (11.72)	6.65 (11.42)	2.26 (1.63)
	*p* -Value	0.302	0.930	0.123	0.103	0.238	0.379

Abbreviation: SD, standard deviation.

Note: Student's *t* -test with 5% significance.

## Discussion

The results of the present study demonstrated that there was no difference comparing the AS with the NS in T0 for all muscles studied, during all facial expressions evaluated, and that a new ocular prosthesis did not influence the electrical activity of the occipitofrontalis, temporal, masseter, and sternocleidomastoid muscles, during all facial expressions evaluated.

Two studies exist in the literature that electromyographically evaluated users of ocular prostheses. 10 11 Goiato et al 10 verified that the restoration of the anophthalmic cavity with an ocular prosthesis promotes an increase in the electrical activity of the orbicular muscles of the eye, restoring part of the muscular tonus and the motor function of the region. 10 The study from Regalo et al 11 verified that the use of an ocular prosthesis does not interfere in the opening and closing of the eyelid, and in addition, the loss of an eye increases the electromyographic activity in the orbicular muscle of the eye. 11 Unfortunately, the present study cannot be compared with these studies, due to differences of methods.

A limitation of this study is the heterogeneity of profiles of the patients (type of eye surgery, length of anophthalmia, and previous use or nonuse of an ocular prosthesis). However, this limitation is justified by the difficulty encountered in recruiting patients with anophthalmia, with homogeneity in terms of the length of the anophthalmia, and that in addition, did not previously use a prosthesis.

The central control of the movements of the face is complex and depends on multiple parallel systems, such as the affective and volitional systems, which are anatomically or functionally segregated, 1 even though these systems are completely independent. This signifies that it is not possible to voluntarily produce a genuinely emotional facial expression. In this way, even with the patients having performed the expressions according to the photographs, these expressions are not equal to the expressions produced when commanded by emotion.

Despite the results of this study not having demonstrated significant differences, an ocular prosthesis can restore the esthetics, prevent deformation of the eyelid, protect the anophthalmic cavity, direct and avoid the accumulation of tear fluid in the cavity, as well as help the tear glands partially recuperate their natural position. 10 11 25 It is also important to emphasize that rehabilitation with an ocular prosthesis is associated with psychosocial improvements, which are capable of positively influencing interpersonal relations. 10 26 Therefore, the use of an ocular prosthesis is recommended for patients with the absence of the ocular globe.

## Conclusion

Eye loss did not affect the EMG of studied muscles when comparing NS with AS (without the use of an ocular prosthesis). The rehabilitation with an ocular prosthesis was not capable of changing the EMG on AS.
